# Activated Complement System’s Impact in Antiphospholipid Syndrome Thrombosis: From Pathophysiology to Treatment

**DOI:** 10.3390/jcm14186672

**Published:** 2025-09-22

**Authors:** Sofia Tagara, Serena Valsami, Eleni Gavriilaki, Elias Kyriakou, Elisavet Grouzi, Paschalis Evangelidis, Paraskevi Karvouni, Georgia Kaiafa, Ioannis Papadakis, Aristarchos Poulis, Eleni Petrou, Marianna Politou, Styliani Kokoris

**Affiliations:** 1 Laboratory of Hematology, General Hospital of Chania “St. George”, 73100 Chania, Greece; sofiatagara@hotmail.com (S.T.); dr.ioannispapadakis@gmail.com (I.P.); 2 Hematology Laboratory and Βlood Bank, Aretaieion Hospital, National and Kapodistrian University of Athens, 11528 Athens, Greece; serenavalsami@yahoo.com (S.V.); mariannapolitou@gmail.com (M.P.); 3 Second Propedeutic Department of Internal Medicine, Aristotle University of Thessaloniki, 54642 Thessaloniki, Greece; elenicelli@yahoo.gr (E.G.); pascevan@auth.gr (P.E.); 4 Laboratory of Hematology and Blood Bank Unit, “Attikon” University General Hospital, School of Medicine, National and Kapodistrian University of Athens, 12462 Athens, Greece; kyriakoue72@gmail.com (E.K.); a_poulis@otenet.gr (A.P.); epekard@yahoo.gr (E.P.); 5 Department of Transfusion Service and Clinical Hemostasis, “Saint Savvas” Oncology Hospital, 11522 Athens, Greece; grouzielisavet@gmail.com; 6 School of Medicine, Aristotle University of Thessaloniki, 54634 Thessaloniki, Greece; paraskeu98@gmail.com; 7 1st Propaedeutic Clinic of Internal Medicine, AHEPA University Hospital, Aristotle University of Thessaloniki, 54634 Thessaloniki, Greece; gdkaiafa@yahoo.gr

**Keywords:** antiphospholipid syndrome, complement, endothelium, inflammation, thrombosis

## Abstract

Antiphospholipid syndrome (APS) is the most common acquired form of thrombophilia and is associated with the presence of antiphospholipid antibodies (aPL) in the patient’s serum. Until now, the “double-hit” hypothesis remains the prevailing theory for APS pathogenesis. According to this model, the presence of aPL (first hit) is insufficient to trigger thrombosis. A secondary event, such as an inflammatory trigger or vascular injury (second hit), is required to initiate immunothrombosis, which ultimately leads to thromboembolism. Although immunothrombosis has a critical role in several mechanisms, such as in defense against pathogens, chronic immune system activation by aPL appears to disrupt its protective function. In the last three decades, the role of the complement system has gained increasing recognition in the pathophysiology of APS. aPL are involved in the dysregulation of multiple components, such as platelets, β2-glycoprotein I, and complement factor H, resulting in excessive activation of the complement system. Thus, the complement system is a key driver of thrombosis in APS and stands as a promising target for the development of future therapeutic strategies. In the current review article, we aim to summarize the ongoing research regarding the role of complement system dysregulation in APS-associated thrombosis development, while recognizing potential therapeutic targets. In the era of precision medicine, more data concerning targeted therapeutics in the field of APS are essential.

## 1. Introduction

Antiphospholipid Syndrome (APS), first described in 1980 by Graham Hughes as Cardiolipin Syndrome, represents the most common form of acquired thrombophilia, associated with the presence of antiphospholipid antibodies (aPL) in patients’ serum [1]. Based on its clinical presentation, it is classified into thrombotic and obstetric types, with the two forms often overlapping [2]. With an incidence of 5 cases per 100,000 per year, APS appears more frequently in young women aged between 20 and 40 years old [3]. In the general population, 10% of thrombotic events are attributed to APS, while patients suffering from it exhibit a 50–80% increased mortality risk, compared to healthy controls [4].

The term aPL refers to a heterogeneous group of autoantibodies that target negatively charged phospholipids of the cell membrane, phospholipid–protein complexes, or proteins bound to phospholipids, such as β2-glycoprotein I (β2GPI), annexin, prothrombin, and vimentin [5]. aPL are present in approximately 1–5% of the general population, with their prevalence increasing with age [5]. Their presence does not necessarily indicate an underlying pathological condition, as transient positivity may occur in the context of viral infections [e.g., human immunodeficiency virus (HIV), Epstein-Barr virus (EBV), hepatitis C virus (HCV)], vaccinations, or malignancies (solid or hematological) [6]. However, the specificity of these aPL associated with infections is different from that observed in systemic autoimmune diseases. For instance, in infectious mononucleosis, the aPL detected are transient, targeting distinct viral epitopes, while in autoimmune disorders, such as APS, they reflect a broader clinical syndrome involving phospholipid-binding plasma proteins [7]. Nonetheless, aPL often precede or coexist with various autoimmune or thrombotic disorders, making careful clinical interpretation essential [4]. Although more than 30 types of antiphospholipid antibodies have been identified, the most clinically relevant and routinely tested in suspected antiphospholipid syndrome are: lupus anticoagulant (LAC), anticardiolipin antibodies (aCL), and anti-β2GPI antibodies [8].

Venous thrombosis is the most frequent clinical manifestation of APS, usually occurring as deep vein thromboembolism (DVT) of the lower extremities [9]. Pulmonary embolism, often the initial disease manifestation, is present in up to 20% of patients with APS [10]. Thrombosis in the arterial circulation, although less common, remains one of the most life-threatening manifestations of the syndrome [11]. Ischemic cerebrovascular events and acute myocardial infarction are the leading causes of death in patients with APS [12]. Additionally, microvascular thrombosis affects approximately 12% of APS patients and requires histopathological confirmation for its diagnosis [13,14]. Most often presenting in the context of catastrophic APS (CAPS), it can potentially involve any organ, with a predilection for the kidneys and skin [15].

Beyond thromboembolic events, thrombotic APS has a broad range of clinical symptoms, not always easily recognized, necessitating a high degree of clinical suspicion and vigilance [16]. Valvular heart disease, also known as Libman-Sacks endocarditis, affects 15–30% of APS patients and can lead to ischemic strokes through aseptic arterial emboli [17]. Central nervous system symptoms frequently occur in APS patients and may be of non-vascular origin. These cover the entire neurological-psychiatric spectrum, including headache, migraine, dementia, epilepsy, myelitis, as well as psychosis and bipolar disorder [18]. Hematological features of the syndrome include thrombocytopenia and autoimmune hemolytic anemia [19]. APS-associated thrombocytopenia occurs in 20–40% of patients, is usually mild to moderate in severity, and is rarely complicated by bleeding [20]. Autoimmune hemolytic anemia is less common (~6.5%), while bone marrow necrosis, described in a few cases of CAPS, is the rarest but very severe hematological manifestation [21].

Until 2023, the clinical diagnosis of thrombotic APS was based on the revised Sydney-Sapporo criteria, requiring the occurrence of arterial or venous thrombosis in either micro- or macrovascular circulation and the presence of persistently positive antiphospholipid antibody titers [22]. Despite the high sensitivity in APS diagnosis of the revised Sydney-Sapporo criteria, some drawbacks have been reported [23]. For instance, non-thrombotic manifestations, as described above, are not considered in APS clinical manifestations. Moreover, some definitions, such as microvascular thrombosis, are not precisely clarified, resulting in heterogeneity among diagnosed APS patients.

The 2023 ACR/EULAR classification criteria address these limitations by clearly defining terms and clinical conditions that previously caused ambiguity in evaluation [24]. They require the presence of one clinical criterion, no more than three years apart from a positive laboratory criterion. If met, the patient can be assessed using additional criteria to calculate a total score that determines classification. The criteria are divided into eight categories: six clinical and two laboratory. Each criterion is weighted from 1 to 7 points based on severity. A total of at least three points from clinical and three points from laboratory categories confirms APS diagnosis. The criteria assign different scores for venous and arterial thrombosis depending on the presence of other thromboembolic risk factors, provide detailed analysis of obstetric morbidity/mortality and microvascular thrombosis, and incorporate non-thrombotic manifestations such as valvular heart disease and thrombocytopenia. Furthermore, laboratory classification criteria have been modified to allow risk stratification based on antibody positivity profiles. Importantly, isolated IgM positivity for aCL and/or anti-β2GPI alone is no longer sufficient for diagnosis. These novel criteria offer greater accuracy for classification and diagnosis and demonstrate higher specificity compared to previous ones [25]. This allows for the creation of more homogeneous subpopulations, facilitating comparative studies and further research. In Table 1, a summary of the 2023 ACR/EULAR classification criteria is given.

Despite the advances that have been made in the diagnosis of APS and the early identification of patients who might experience a higher risk of thromboembolic events, more data regarding the pathogenesis of this syndrome are crucial for the development of novel and personalized therapeutics in the field. In this review, we aim to summarize the published data regarding the role of complement system activation in APS thrombosis, discussing also potential therapeutic targets.

## 2. An Overview of APS Pathophysiology

Pathophysiology of APS remains an active field of research that has revealed multiple contributing mechanisms. The most widely accepted model explaining APS pathogenesis is the “two-hit hypothesis” [26]. According to this model, the first hit involves the presence of aPL on the membranes of target cells, which promotes the development of a prothrombotic state. Anti-β2GPI antibodies reduce the levels of β2GPI and impair its antithrombotic function. Additionally, factors such as reactive oxygen species (ROS) influence its conformation and promote its dimerization on the cell membrane surface. β2GPI dimers exhibit significantly increased affinity for aPL. The anti-β2GPI/β2GPI complex binds to cellular receptors and promotes an inflammatory response, which, following a second hit, can lead to thrombosis [27]. The second hit, often clinically silent, is associated with vascular injury, venous stasis, or inflammation and establishes conditions conducive to coagulation activation. This theory is supported by animal models, where injection of aPL alone does not induce thrombosis, but thrombosis occurs when vascular homeostasis is disturbed—for example, by vascular injury, altered blood flow, or lipopolysaccharide administration [28].

A hallmark feature in all APS patients is the persistent and excessive inflammatory response induced by aPL, which activates the coagulation system and promotes thrombosis, a process known as immunothrombosis [28]. Under physiological conditions, immunothrombosis serves as a protective host mechanism, helping to trap and neutralize pathogens locally through clot formation. However, in conditions of persistent immune stimulation, as seen in APS, it may become pathological, leading to serious thromboembolic complications. The formulated thrombus interacts with immune cells, the complement system, and endothelium, thereby amplifying the immune response in a process called thrombo-inflammation, and, thus, sustaining a vicious cycle of ongoing over-activation [29]. aPL have multiple cellular targets and mechanisms of action, through which they can trigger immunothrombosis and cause damage to various vital organs.

### 2.1. Role of Endothelial Cell Activation

aPL can activate endothelial cells by binding to surface receptors such as apolipoprotein E receptor 2 (ApoER2), annexin 2, and Toll-like receptor 4 (TLR4) [30]. These interactions activate the nuclear factor kappa-light-chain-enhancer of activated B cells (NF-κB) and p38 mitogen-activated protein kinase (MAPK) signaling pathways via phosphorylation, leading to increased transcription of pro-inflammatory cytokines and the expression of adhesion molecules, including E-selectin, intercellular adhesion molecule 1 (ICAM-1), and vascular cell adhesion molecule 1 (VCAM-1) [31]. This proinflammatory state is accompanied by the secretion of tissue factor (TF) and interleukin-6 (IL-6), further enhancing the prothrombotic environment. In addition, activation of p38 MAPK induces the production of ROS, which depletes nitric oxide (NO) production, an endothelial-derived vasodilator and inhibitor of platelet aggregation. Concurrently, the loss of thrombomodulin and heparan sulfates on the endothelial surface compromises its antithrombotic properties, collectively promoting a procoagulant state [28].

Additionally, based on recent findings, endothelial cells can be directly activated by anti-β2-GPI antibodies via a LRP6/β-catenin–dependent pathway. Anti-β2-GPI binding induces phosphorylation of LRP6 (Low-Density Lipoprotein Receptor-Related Protein 6) and subsequent activation of β-catenin, leading to TF expression. Importantly, for this pathway, the structural integrity of lipid rafts is essential [32].

### 2.2. Role of Monocytes

aPL are known to trigger the activation of monocytes, which is marked by increased adhesion to the endothelial cells via ICAM-1 and VCAM-1 [33]. Anti-β2GPI antibodies stimulate the expression of TF and promote the release of tumor necrosis factor-α (TNF-α) and interleukin-1β (IL-1β) by monocytes, primarily through the activation of Toll-like receptor 9 (TLR9). In addition, these antibodies enhance TLR4-mediated signaling pathways and increase the expression of very late antigen-4 (VLA-4), an integrin critical for monocyte adhesion [34]. Monocytes from patients with antiphospholipid syndrome exhibit elevated levels of vascular endothelial growth factor (VEGF) and its receptor Flt-1, which seem to play a role in further TF expression. Simultaneously, they activate protease-activated receptors (PARs), which are integral in coagulation and complement system activation.

### 2.3. Neutrophil Extracellular Traps (NETs)

**Figure 1:**
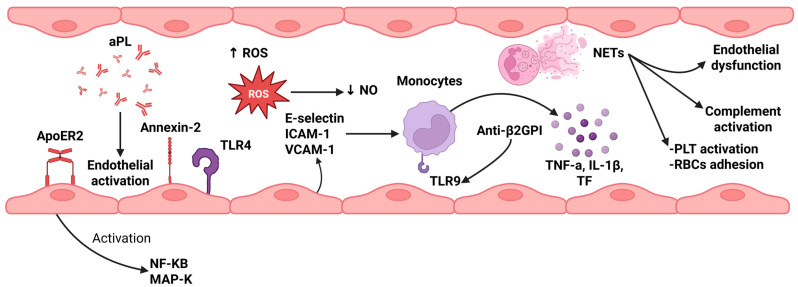
An overview of endothelial activation, monocytes, and NETs’ role in APS-thrombosis pathogenesis. Several mechanisms are implicated in the development of thrombosis in APS patients. aPL lead to activation of endothelial cells, via several receptors, such as ApoER2, Annexin-2, and TLR4, which result in the upstream regulation of NF-KB and MAP-K signaling pathways. Endothelial activation is associated with the release of ROS, which deplete NO production (NO acts as a vasodilator) and of E-selection, ICAM-1, and VCAM-1, molecules responsible for the migration of inflammatory cells, including monocytes. Moreover, monocytes can be activated by anti-β2GPI antibodies, via TLR9, and, thus, release both pro-inflammatory cytokines, such as TNF and IL-1Β, and TF, which activate the extrinsic coagulation pathway. NETs can also lead to PLT activation, increased RBC adhesion, endothelial dysfunction, and complement activation, which further reinforce the vicious cycle between thrombosis and inflammation. Created in BioRender. Evangelidis, P. (2025). https://biorender.com/vfbsik6 (accessed on 22 August 2025). Anti-β2GPI: anti-β2 Glycoprotein I antibodies; aPL: antiphospholipid antibodies; APS: antiphospholipid syndrome; ICAM-1: intercellular adhesion molecule-1; IL-1β: interleukin-1 beta; MAP-K: mitogen-activated protein kinase; NETs: neutrophil extracellular traps; NF-KB: nuclear factor kappa-light-chain-enhancer of activated B cells; NO: nitric oxide; PLT: platelets; RBCs: red blood cells; ROS: reactive oxygen species; TF: tissue factor; TLR4: toll-like receptor 4; TLR9: toll-like receptor 9; TNF-α: tumor necrosis factor alpha; VCAM-1: vascular cell adhesion molecule-1.

NETs contribute to inflammation and immunothrombosis and can ultimately lead to vascular thrombosis [35]. Their prothrombotic activity is attributed to their ability to promote platelet activation and red blood cell adhesion, as well as to the inactivation of anticoagulant factors, such as antithrombin, tissue factor pathway inhibitor (TFPI), and protein C [36]. In the past decade, a significant link has been identified between aPL and NET formation. Specifically, anti-β2GPI antibodies have been shown to promote NET release, especially in triple-positive patients [36]. Elevated circulating NET levels have also been reported in APS patients, even in the absence of clinically evident thrombosis, along with signs of impaired NET clearance [28]. Recent studies suggest that NETs play a complex role in the pathogenesis of antiphospholipid syndrome. They act as a source of autoantigens, activate the complement and coagulation systems, stimulate inflammatory cells, and contribute to endothelial dysfunction, all of which facilitate thrombus formation [36]. In Figure 1, an overview of endothelial activation, monocytes, and NETs’ role in APS-thrombosis pathogenesis is provided.

### 2.4. Platelet Activation

The anti-β2GPI/β2GPI complex is thought to be crucial in platelet activation by engaging receptors such as Toll-like receptors, ApoER2, and GPIbα [37]. These interactions activate intracellular signaling pathways, including the p38 MAPK and phosphatidylinositol 3-kinase (PI3K)/protein kinase B (AKT)/mechanistic target of rapamycin (mTOR) pathways, which contribute to platelet adhesion and aggregation [38]. This process is further enhanced by the elevated release of von Willebrand factor (vWF) and the reduced activity of ADAMTS-13, both of which are commonly observed in APS patients [39]. Additionally, increased levels of platelet-derived microparticles, which possess procoagulant properties, are frequently found in the circulation of these patients, as a result of ongoing, low-grade platelet activation [40]. The mild thrombocytopenia often seen in APS may be partly attributed to this persistent activation and the accelerated clearance of platelets.

### 2.5. Dysregulation of Coagulation and Fibrinolytic Systems

The impact of aPL has also been studied in coagulation and fibrinolytic systems [41]. In patients with APS, elevated levels of coagulation factor XI have been observed, which promote the activation of factors IX and X, leading to the formation of the prothrombinase complex. Furthermore, aPL have been shown to affect factor XII, by reducing both its antigen levels and activity, impairing plasminogen activation and inhibiting fibrinolysis. At the same time, they enhance factor XIII activity, resulting in the formation of more stable fibrin clots that are resistant to degradation by plasmin [41]. Additionally, aPL targeting factors IXa, Xa, and thrombin have been identified. These antibodies interfere with the inactivation of these factors by antithrombin [42,43]. Moreover, antibodies against activated protein C hinder their ability to regulate the tenase and prothrombinase complexes. In patients with positive LA, reduced activity of TFPI has also been reported [28]. Disturbances in fibrinolytic mechanisms have also been noted in APS, as aPL may bind to fibrinolytic proteins such as plasmin, annexin A2, and tissue plasminogen activator (tPA), thereby impairing their function. Taken together, these findings suggest that APS is characterized by a profound disruption of coagulation homeostasis. Excessive thrombin generation, formation of more resistant clots, and impaired fibrinolysis contribute to a prothrombotic environment.

## 3. Complement System in APS

Complement activation by aPL has been shown to occur through both the classical and alternative pathways [44]. Elevated levels of complement activation markers have been observed in patients with aPL and thrombosis, while other studies link APS to reduced levels of C3 and C4 [45]. A recent study of patients with primary APS also reports lower levels of complement inhibitors [46]. In clinical practice, monoclonal antibodies targeting C5 have been used with positive outcomes in patients with APS characterized by recurrent thrombotic events and CAPS cases, not responding to standard therapy [47]. These findings support the critical role of complement in the pathogenesis of the syndrome.

### 3.1. An Overview of Complement Biology

The complement system is a key component of the innate immune system, comprising over 50 plasma or membrane-bound proteins that trigger immune responses against pathogens, neoplastic cells, and other foreign substances [48]. It was discovered in 1896 by Jules Bordet and was initially described as a heat-labile “complementary” factor to the action of immunoglobulins [49]. Complement proteins, primarily secreted by the liver, constitute approximately 15% of plasma globulins and have an average concentration of ~3 mg/mL. They become locally activated, following exposure to pathogens, and act as pattern recognition receptors (PRRs) [48].

Upon pathogen recognition, complement components are hierarchically activated, leading to opsonization by C3b and C4b, and the generation of pro-inflammatory mediators C3a and C5a. The formation of the membrane attack complex (MAC) ultimately results in the lysis of the foreign cell. There are three distinct pathways of complement activation: the classical pathway, the lectin pathway, and the alternative pathway. All converge at a common terminal step—the formation of C5b.

Regardless of the activation pathway, the complement system defends the host through three primary mechanisms: (1) direct lysis and destruction of the pathogen via the membrane attack complex (MAC), (2) induction of an inflammatory response through the action of anaphylatoxins, and (3) opsonization of pathogenic targets by complement opsonins such as C3b, C3bi, and C4b, leading to their clearance from circulation.

Uncontrolled complement activation, particularly its continuous amplification via the alternative pathway, may have detrimental effects on the host [50]. Therefore, a regulatory system is required to ensure that complement activity occurs strictly under immune defense conditions, without targeting the host’s normal cells. This regulation is achieved through two levels of control. The first level modulates the production and activity of the convertases, while the second level regulates the formation of the membrane attack complex (MAC).

**Figure 2:**
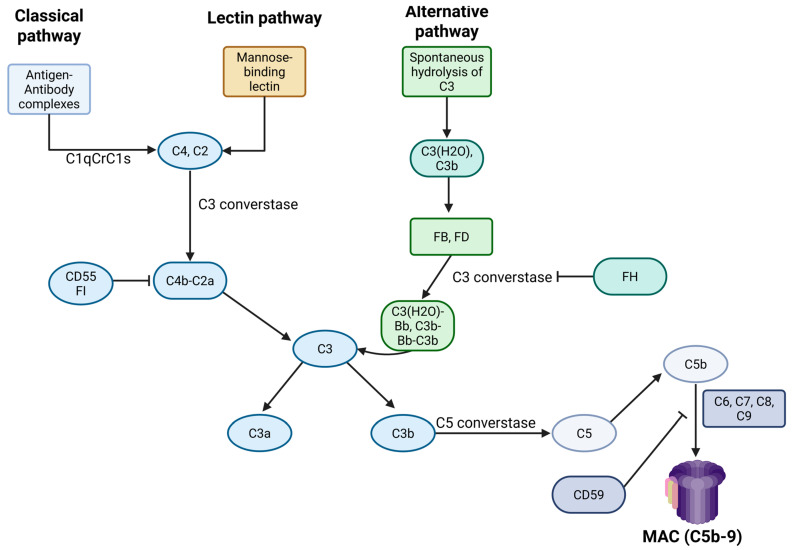
An overview of complement system biology. Created in BioRender. Evangelidis, P. (2025) https://biorender.com/r66qg8z (accessed on 7 August 2025), FH: factor H; FI: factor I; MAC: membrane attack complex.

Originally, its role was thought to be limited to innate immunity, including the recognition and destruction of pathogens. However, subsequent research has highlighted its importance and interactions with cells of the adaptive immune system. Today, it is well known that the complement system also participates in processes beyond immunity and interacts with other biological pathways, such as the coagulation cascade [51]. Meanwhile, its role in the pathogenesis of various diseases associated with thromboembolism, such as paroxysmal nocturnal hemoglobinuria (PNH) and APS, has become more widely acknowledged [52,53]. In Figure 2, an overview of complement system biology is given.

### 3.2. Complement System Activation in APS

The contribution of the complement system in thrombotic APS has been increasingly recognized over the past three decades [47]. Several studies have demonstrated that complement activation is essential for thrombosis in patients with APS, and it is now universally accepted that its involvement in the syndrome’s pathophysiology is fundamental.

In 2005, Pierangeli et al. investigated the role of the complement system in thrombus formation using C3 and C5 knockout mice, which were treated with aPL from APS patients [54]. After inducing vascular injury, they observed that the C3^−/−^ and C5^−/−^ mice exhibited reduced leukocyte adhesion to the injured endothelium and formed significantly smaller thrombi compared to wild-type controls. Additionally, they showed that C5 inhibition with an anti-C5 antibody reduced thrombus formation in C3^+^/C5^+^ mice. These findings provided compelling evidence that the activation of C3, and particularly C5, is essential for the thrombotic manifestations of APS.

In parallel, Fischetti et al. (2005) studied animals that were injected with anti-β2GPI antibodies [55]. Following intraperitoneal administration of bacterial polysaccharide, they found that the anti-β2GPI-negative mice did not develop thrombi, whereas histopathological analysis of the vessels in the aPL-positive animals revealed C3 and C9 deposition along with anti-β2GPI antibodies. They also observed that the C6-deficient mice phenotype or the administration of a C5 inhibitor prevented thrombosis, leading them to conclude that activation of the terminal complement pathway plays a crucial thrombogenic role in APS, although it requires an initiating stimulus. Similarly, Carrera Marin et al. (2012) observed that C6-deficient mice, when administered aPL, developed smaller thrombi and exhibited significantly lower TF levels compared to their C6-sufficient counterparts [56].

Alongside research in animal models, studies in patients with APS further reinforce the role of the complement system activation in the syndrome’s pathogenesis. In a study by Davis et al., higher levels of C5b-9 were found in aPL-positive patients with ischemic cerebral events compared to aPL-negative patients [57]. In a study involving 36 APS patients, Oku et al. (2009) observed lower levels of C3 and C4, alongside increased C3a and C4a levels, suggesting enhanced consumption of the former molecules [45]. Later, Breen et al. (2012) demonstrated increased complement activation in APS patients, identifying higher levels of Bb and C3a-desArg, which were notably elevated in patients with high-risk aPL [58]. In 2016, Meroni et al. reported a case of a patient with APS and arterial thrombosis, where immunofluorescence revealed the presence of β2GPI IgG complexes and deposition of C1q, C4, and C3 on the thrombotic endothelium, findings indirectly consistent with activation of the classical complement pathway [59].

According to Lonati et al. (2019), the deposition of C4d on platelets and erythrocytes was found to be primarily associated with the presence of IgG anti-β2GPI and aCL antibodies, and the levels of C4d correlated with the concentration of these antibodies [60]. Chaturvedi et al. (2020) used the modified Ham test (mHam) to compare complement activation levels in patients with systemic lupus erythematosus (SLE), APS, and CAPS. They reported higher activation rates in APS patients (35.6%) compared to SLE patients (6.8%), while in CAPS patients, it reached 85.7%. Furthermore, maintenance of a positive mHam test for over a year was associated with higher rates of recurrent thrombosis, even under anticoagulant therapy [61]. mHam test was initially developed by Gavriilaki et al. in 2015 for the differential diagnosis of atypical hemolytic uremic syndrome, a complement-mediated disorder, from other thrombotic microangiopathies [62].

### 3.3. Underlying Mechanisms of Complement Dysregulation in APS

Although it is evident, based on the above-described data, that complement’s system involvement in APS thrombosis is unquestioned, the precise mechanism by which aPL initiates complement activation has yet to be completely clarified. Possible activation pathways and processes that enhance their activity that have been described are the following:

#### 3.3.1. Activation Through the Classical Pathway

The hypothesis that complement activation via the classical pathway results from immune complexes formed by aPL, although reasonable, remains debatable. This is because the aPL associated with thrombosis are predominantly of the IgG2 subclass, which exhibits limited capacity to activate the complement system [63]. Nevertheless, studies have demonstrated that serum from patients with aPL can induce deposition of C1q and C4d on heterologous platelets [64]. The predominant theory proposed for this is that aPL bind to platelet membranes, forming immune complexes which, depending on their subclass, can initiate the classical complement pathway [64]. Furthermore, C1q has been shown to bind directly to negatively charged phospholipids on apoptotic cells, independently of immune complexes, thereby triggering complement activation [65]. It is likely, however, that additional mechanisms are also involved in this process.

#### 3.3.2. The Contribution of Platelets

Activated platelets by aPLs also seem to play a key role in complement activation by providing a favorable surface for its initiation [66]. These platelets express P-selectin and gC1qR on their membrane, while their α-granules release chondroitin sulfate. Chondroitin sulfate, a proteoglycan sequence, acts as a pattern recognition molecule (PRM) capable of activating the complement system via the C1q component. The binding of C1q to chondroitin sulfate facilitates its interaction with gC1qR at the IgG-binding region, ultimately leading to classical pathway activation in the absence of immune complexes [66].

The presence of P-selectin on the platelet surface facilitates the binding of hydrolyzed C3b (C3(H_2_O)), thereby contributing to the activation of the alternative complement pathway [67]. C3b is stabilized by properdin and can lead to the formation of the C3 convertase, promoting a positive feedback loop through continuous C3b generation. Additionally, platelets can activate the alternative pathway via extracellular phosphorylation of C3. ATP and Ca^2+^ released from dense granules mediate C3 phosphorylation, resulting in increased levels of phosphorylated C3b, which is more resistant to inactivation by Factor I. Phosphorylated C3b also displays enhanced affinity for CR1 receptors and, upon binding to cell membranes, may further promote activation of the alternative pathway [66].

#### 3.3.3. The Significance of β2GPI

Given that β2GPI represents the primary antigenic target of aPL, it has become a central focus of research. β2GPI can bind to various molecules, including phospholipids, lipopolysaccharides, complement component C3, coagulation factor XII, platelet proteins GP1b and platelet factor 4 (PF4), vWF, and plasminogen, among others [41]. These interactions allow β2GPI to influence coagulation, immune responses, and angiogenesis [41]. In their study, Gropp et al. (2020) explored the role of β2GPI and demonstrated its importance as a key modulator of complement activation [68]. β2GPI binds to apoptotic cells, recruiting C3 and inducing conformational changes that facilitate its cleavage by Factor H. This process halts complement activation at the C3 level, promoting opsonization of apoptotic cells and their removal by macrophages, while simultaneously inhibiting the assembly of convertases, thereby blocking the activation of both the classical and alternative complement pathways.

Moreover, in hemostasis, it demonstrates a procoagulant role by binding to thrombin and preventing its inactivation by antithrombin, thereby sustaining thrombin activity [69]. Furthermore, it inhibits the inactivation of activated factor V by activated protein C, contributing to the amplification of the coagulation cascade. It also suppresses fibrinolysis that is dependent on factor XII. Despite these procoagulant functions, it simultaneously exhibits antithrombotic properties that predominate. Notably, it prevents the inactivation of protein S, a crucial cofactor in the anticoagulant pathway, and exerts antiplatelet effects by decreasing platelet adhesion and aggregation. This is achieved through the inhibition of the interaction between platelets and vWF, which is essential for platelet adhesion under high shear conditions.

Anti-β2GPI antibodies act by inhibiting these regulatory functions of β2GPI. Consequently, they promote the already exaggerated activation of both the coagulation cascade and the complement system in APS.

#### 3.3.4. The Role of Factor H

Factor H belongs to a family of regulatory proteins called Factor H-related proteins, which also includes FHR1, FHR5, and FH-like protein 1 (FHL-1). Factor H is a central regulator of the alternative complement pathway. Beyond serving as a cofactor for Factor I and DAF, FH also competes with Factor B to bind C3b, blocking C3 convertase formation [70]. In addition, it interacts with coagulation factors, inhibiting their activity [70]. Factor H exhibits structural and functional similarities in common with β2GPI. Both molecules bind to negatively charged membrane phospholipids and inhibit the activation of FXIIa and FXIa, thereby preventing the initiation of the coagulation cascade [71]. Low levels of Factor H have been reported in patients with APS. Nakamura et al. (2018) compared patients with primary or secondary APS to patients with SLE and found significantly reduced FH levels in the APS group [46]. This reduction was not associated with the type or titer of aPL, nor with the presence of anti-FH antibodies.

Other research groups have reported the detection of anti-FH antibodies in patients with APS, which inhibit Factor H function against complement activation and impair its antithrombotic properties. Zadura et al. studied two populations of APS patients in comparison with individuals with DVT and observed high titers of anti-FH antibodies in the APS group [72]. This finding was also associated with a higher incidence of venous thrombosis and non-thrombotic cardiac manifestations of the syndrome. In any case, low levels of Factor H appear to contribute to enhanced activation of the alternative complement pathway [46].

In addition to anti-FH antibodies, antibodies against NETs and C1q have also been implicated in complement activation in APS. The increased formation of NETs observed in APS may promote the generation of IgM anti-NET antibodies. These antibodies are thought to stabilize the structure of NETs, promoting the recruitment and activation of complement proteins, including C3, C1q, factor B, and properdin [73]. Evidence indicates that anti-C1q antibodies promote complement activation in primary APS, and their elevated levels have been associated with more severe and refractory clinical presentations [65].

#### 3.3.5. The Impact of Mutations

Genetic modifications or polymorphisms in complement-related genes have been associated with enhanced activation of the complement system [44]. These alterations may impair its regulation or potentiate its activity on cellular membranes. They are more often in patients with CAPS and represent an additional critical “hit” in disease pathogenesis. The *CFHR1* and *CFHR3* genes encode the FHR-1 and FHR-3 proteins, which regulate Factor H function by competing with it at binding sites. Deletions in these genes, which represent a genetic variant found in approximately 2–5% of the general population, have been reported in homozygosity in patients with CAPS. Their presence has been linked to the development of anti-FH antibodies and increased complement activation [44]. Other genetic defects that result in uncontrolled complement activity include point mutations in the *CR1* gene (*CR1 V1675L*), the *CFHR4* gene (*CFHR4 R287H*), as well as mutations affecting the *THBD* gene encoding thrombomodulin (*THBD P501L*) [61]

## 4. Complement’s Interplay with Thrombosis

Once activated by aPL, complement fragments enhance immunothrombosis through multiple pathways, as they interact with procoagulant factors through various mechanisms [74]. Specifically, they:

(a) modify cell membranes by inducing the exposure of negatively charged phospholipids, which are essential for the initiation of coagulation.
(b) activate platelets and neutrophils.
(c) induce the expression of TF by various cell types.
(d) disrupt the fibrinolytic process.

Platelets express specific surface receptors for anaphylatoxins, including C3aR for C3a, and C5aR1 and C5L2 for C5a [75]. Engagement of these receptors by their respective ligands triggers platelet activation and promotes the surface translocation of gC1qR (the receptor for complement component C1q). Βinding of C1q to its receptor induces conformational changes in platelet glycoprotein GpIIb/IIIa, enhancing platelet adhesion and aggregation. Simultaneously, P-selectin expression facilitates leukocyte recruitment to the site of activation. C4a, an anaphylatoxin produced via the classical or lectin complement pathways, also contributes to platelet activation by interacting with protease-activated receptors PAR1 and PAR4 on the platelet surface [75].

Moreover, anaphylatoxins C3a and C5a exert significant effects on endothelial cells. Through engagement of their respective receptors, they induce the expression of vWF, while downregulating the expression of thrombomodulin [76]. C4a, acting through protease-activated receptors PAR1 and PAR4, also contributes to endothelial activation. Together, these anaphylatoxins trigger intracellular signaling cascades that result in cytoskeletal rearrangement and compromise of the endothelial barrier integrity [75]. Furthermore, C5a–antibody complexes contribute to the removal of heparan sulfate from the endothelial surface. This process impairs the function of antithrombin, which relies on heparan sulfate for its anticoagulant activity [74].

Complement activation—particularly the generation of C5a—plays a major role in promoting coagulation by inducing TF expression on monocytes and neutrophils, thereby facilitating initiation of the extrinsic coagulation pathway [74]. In addition, anaphylatoxins modulate cytokine production, leading to increased levels of TNF-α, which further enhances TF expression on monocytes, and interleukin-6 (IL-6), which contributes to platelet activation. Mast cells, through the production of heparin and tissue plasminogen activator (tPA), help regulate the activation of coagulation, preventing excessive thrombotic activity. C5a acts on mast cells, stimulating the expression of plasminogen activator inhibitor-1 (PAI-1) at levels higher than those of tPA. A similar effect is observed in basophils, leading to a reduction in fibrinolysis. Overall, anaphylatoxins promote a procoagulant state by directly activating coagulation factors and compromising the anticoagulant properties of the endothelium [74].

Although data remain limited, the membrane attack complex (MAC, C5b-9) appears to exert prothrombotic effects by altering cellular membranes and influencing thrombus composition. Under normal conditions, MAC formation on platelets is inhibited by regulatory proteins such as CD55 and CD59. However, once formed, MAC can activate platelets, leading to the release of granule contents and promotion of a procoagulant phenotype.

On endothelial cells, MAC disrupts barrier integrity, increasing vascular permeability and exposing subendothelial TF, which can initiate the extrinsic coagulation pathway [77]. Additionally, activation of the terminal complement pathway results in the generation of platelet- and endothelial-derived microparticles. These vesicles are rich in TF and display negatively charged phospholipid surfaces, which serve as catalytic platforms for coagulation complex assembly and amplification of thrombin generation.

C5b-9 (MAC) also plays a critical role in neutrophil activation, triggering the formation of neutrophil extracellular traps [76]. Their activation by MAC, as well as by other complement components, leads to the release of proteolytic enzymes (such as elastase, myeloperoxidase, and cathepsins), ROS, proinflammatory cytokines, and PF4. These molecules contribute to platelet activation and enhance the activity of coagulation factors V, VIII, and X, while inhibiting natural anticoagulants like antithrombin, TF pathway inhibitor, and thrombomodulin. Additionally, NETs serve as procoagulant platforms due to their negatively charged surface, which facilitates the activation of the contact pathway through factors XI, XII, and kallikrein. Additionally, the release of TF and microparticles from activated neutrophils further amplifies the thrombotic response. Histones H3 and H4, components of the NETs, play an important role in inhibiting the anticoagulant effect by inactivating antithrombin and protein C [76].

**Figure 3:**
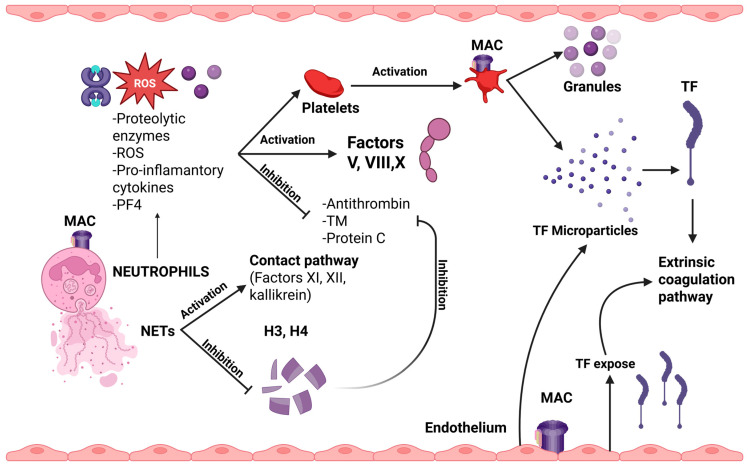
Impact of MAC formulation on neutrophils, platelets, and endothelial cells. Formulation of MAC (C5b-9) on the cell surface of neutrophils leads to the release of proteolytic enzymes, ROS, pro-inflammatory cytokines, and PF4, which can activate platelets and factors V, VIII, and X, while inhibiting natural anticoagulants, such as antithrombin, TM, and protein C. Moreover, NETs production contributes to the activation of the contact pathway factors and further inhibition of the above-described anticoagulants. Platelets’ activation from MAC leads to the release of granules and TF microparticles, activating the extrinsic coagulation pathway. Additionally, this pathway can be activated by endothelial-TF-related production. Thus, a cycle between complement system activation and coagulation cascade is established. Created in BioRender. Evangelidis, P. (2025) https://biorender.com/a7rn9oe (accessed on 7 August 2025), H3: histone 3; H4: histone 4; MAC: membrane attack complex; NETs: neutrophil extracellular traps; PF4: platelet factor 4; ROS: reactive oxygen; TF: tissue factor; TM: thrombomodulin.

In summary, complement activation mediated by aPL enhances the processes of immunothrombosis, resulting in a self-sustaining feedback loop that is difficult to interrupt. Recurrent thrombosis in APS patients has been linked to increased complement activity. These patients show a positive mHam test at double the rate, compared to those without recurrent events. A similar association has been observed with the presence of anti-factor H antibodies, while elevated concentrations of C5a and C5b-9 have been detected in patients with refractory APS. These findings may be utilized to identify patients at higher risk and aid in guiding them toward the most appropriate therapeutic approach [78]. In Figure 3, the impact of MAC formulation on neutrophils, platelets, and endothelial cells is presented.

## 5. APS Treatment Approach

### 5.1. Current Treatment

Considering the complex nature of the syndrome, the development of a targeted and consistently effective treatment remains a significant challenge for APS patients. Current management is predominantly symptomatic and primarily based on secondary prevention through indefinite anticoagulation. The standard of care for patients with APS is long-term use of vitamin K antagonists (VKAs). According to the European Medicines Agency (EMA) guidelines, which have been broadly accepted, the use of direct oral anticoagulants (DOACs) in APS is not recommended, although it is not formally contraindicated [2,79].

Treatment with vitamin K antagonists is initiated with bridging anticoagulation using low molecular weight heparin (LMWH), which is continued until a therapeutic international normalized ratio (INR) is achieved [79]. The site of the thrombotic event—whether venous or arterial—determines the appropriate target INR as well as subsequent therapeutic strategies.

For a first episode of venous thrombosis, the recommended initial INR target is 2–3, as no additional benefit has been demonstrated with higher values [79]. In cases where patients are unable to maintain therapeutic INR levels or have contraindications to VKAs, the use of DOACs may be considered under specific circumstances. Treatment duration typically ranges from 3 to 6 months for provoked thrombosis, whereas unprovoked thrombosis generally necessitates extended or lifelong anticoagulation. Regarding the first arterial thrombotic episode, guidelines recommend initiation of therapy with VKAs rather than antiplatelet monotherapy, given the substantially elevated risk of recurrence with aspirin alone. The therapeutic INR target for arterial thrombosis usually lies between 3–4, with individualization based on patient-specific bleeding and thrombotic risks. Alternatively, an INR target of 2–3 combined with antiplatelet agents may be employed [79,80].

For patients experiencing recurrent thrombosis despite adequate VKA therapy, assessment of treatment adherence is essential; upon confirmation, therapeutic adjustments may include the addition of low-dose aspirin, escalation of the INR target, or transition to low molecular weight heparin [79]. Importantly, DOACs are not recommended for arterial thrombosis management in APS.

The efficacy of DOACs in the management of antiphospholipid syndrome remains controversial. Rivaroxaban and apixaban, both factor Xa inhibitors, are the most extensively studied agents in this field. Several studies have reported an increased risk of thrombosis in patients treated with these agents. Notably, the TRAPS trial, which compared rivaroxaban to warfarin in 120 patients, all with triple aPL positivity, demonstrated a significantly higher recurrence rate in the rivaroxaban arm (33.3% vs. 5.7%) [81]. The ASTRO-APS study, investigating the use of apixaban for secondary prevention in APS, enrolled 47 patients; however, the trial was prematurely terminated due to increased rates of arterial thrombosis in the apixaban group [82]. These findings suggest that DOACs are associated with a higher risk of thrombotic complications, particularly arterial events, in APS [82]. It appears that Xa inhibition alone may be insufficient to prevent thrombosis in APS, although further data are expected from the ongoing RISAPS trial, which compares high-dose rivaroxaban (15 mg twice daily) to warfarin in APS patients with ischemic stroke [83]. Additional clinical studies are also needed to evaluate the efficacy of dabigatran, a direct thrombin inhibitor, in secondary prevention of APS, as it may offer improved outcomes [84]. So far, most scientific societies remain cautious about prescribing DOACs in patients with APS. The European Society of Cardiology and the American Society of Hematology, in their 2019 and 2020 guidelines respectively, recommend against the use of DOACs in APS [85,86]. However, the European Alliance of Associations for Rheumatology (EULAR, 2019) and the British Society for Haematology (2020) guidelines allow the use of DOACs in patients with venous thrombosis who do not exhibit triple positivity [79,87].

Adjunctive therapies include hydroxychloroquine, statins, and vitamin D. Hydroxychloroquine, an antimalarial agent, widely used in the treatment of SLE and rheumatoid arthritis, exhibits immunomodulatory and antithrombotic effects [88,89,90]. In APS, it has been shown to inhibit antiphospholipid antibody-mediated disruption of Annexin A5, thereby preserving endothelial integrity. Moreover, it decreases platelet activation and aggregation by targeting GPIIb/IIIa receptors and mitigates inflammatory responses by reducing β2GPI binding to cell membranes and subsequent antigen exposure [89,90]. Statins, such as fluvastatin and simvastatin, are HMG-CoA reductase inhibitors commonly prescribed for hyperlipidemia. Their anti-inflammatory, antithrombotic, and immunomodulatory properties have supported their use in APS treatment [91]. These agents interfere with antiphospholipid antibody effects on endothelial cells by downregulating adhesion molecule expression and lowering pro-inflammatory cytokines including IL-1β, VEGF, and TNF-α. They further inhibit TF expression by activated monocytes, which contributes to their antithrombotic properties. Low levels of Vitamin D, a fat-soluble vitamin vital for immune system function, have been linked to increased thrombosis risk in APS patients [92]. This association is attributed to the diminished anti-inflammatory and thromboprotective effects of vitamin D. It is believed that vitamin D reduces endothelial activation by suppressing angiogenic factors. Moreover, it inhibits TF expression in cells and blocks the activation of TLR4, leading to decreased inflammatory responses [89,93].

Second-line therapies for refractory APS include plasma exchange and corticosteroids, along with medications capable of reducing, but not eliminating, aPL levels. Intravenous immunoglobulin (IVIg) acts by binding to Fc receptors, thereby neutralizing antiphospholipid antibodies and promoting their clearance, while also diminishing complement activation. The potential benefit of IVIg was emphasized by Tenti et al. (2016), who suggested that IVIg may reduce thrombotic events in patients resistant to standard treatment [94]. Limited data exist regarding the use of rituximab in APS. In a small series of patients with secondary APS associated with SLE, administration of rituximab reduced thrombotic episodes in 80% of patients [94]. The RITAPS study by Erkan et al. (2013) included 19 patients and was designed to assess the safety of rituximab in APS patients and its efficacy in non-thrombotic manifestations of the syndrome [95]. The results showed that although rituximab does not decrease antibody titers, it improves non-criteria manifestations such as thrombocytopenia, skin ulcers, nephropathy, and valvular heart disease.

Complement inhibitors have emerged as a promising therapeutic option for patients with refractory APS, particularly in those exhibiting increased complement activity [96,97]. Eculizumab is a monoclonal antibody directed against complement component C5, and by binding to it, prevents the formation of the MAC. Its use has been associated with clinical improvement in cases of thrombotic microangiopathy that are resistant to glucocorticoids, anticoagulants, and immunosuppressants [97]. Eculizumab has also shown efficacy in the treatment of CAPS, where conventional approaches fail [98]. However, these data come from single case reports or case series with a small number of participants. Thus, more studies are essential to examine the effectiveness of complement inhibition in CAPS and selected APS cases. Multicenter collaboration is essential in this field.

### 5.2. Emerging Therapies

Considering the complex pathogenesis and clinical variability of APS, it becomes clear that inhibiting a single pathogenic pathway is unlikely to be sufficient for effective disease control. Although there is significant scientific interest in exploring and implementing additional therapeutic approaches, the rarity of APS refractory cases remains a major barrier to the design of large, robust clinical trials aimed at evaluating the efficacy of novel or existing treatments.

Among the potential future therapeutic approaches for APS is the targeted suppression of B-cell and plasma cell activity. Agents such as ofatumumab and obinutuzumab—monoclonal antibodies directed against CD20—exert B-cell depleting effects, like Rituximab, can be used for this aim. Another promising agent is belimumab, a monoclonal antibody targeting the B-cell activating factor (BAFF), which impairs B-cell survival and their differentiation into antibody-secreting plasma cells by blocking BAFF signaling. However, there is no strong clinical evidence concerning the use of B-cell targeted therapies in patients with APS. Thus, there is an unmet need for further research with well-designed controlled trials. Daratumumab, an anti-CD38 monoclonal antibody currently used in plasma cell dyscrasias, suppresses plasma cell expansion and is being investigated in APS. The ongoing DAREAPS trial is evaluating its safety in patients with primary APS, with study completion anticipated in 2027 [99]. It remains to be determined whether these targeted therapies can effectively reduce antiphospholipid antibody levels and provide clinical benefit in APS [93,100].

Current treatment of APS is primarily focused on symptomatic control of thrombotic events, while the use of anti-inflammatory agents is generally reserved for more complex or refractory cases, often with limited success. Persistent or residual inflammatory responses may play a key role in triggering thrombotic relapses and may contribute to treatment-resistant forms of the disease. Targeting immunothrombosis through specific therapeutic agents presents a complex challenge. Nonetheless, such an approach, based on disease pathophysiology rather than symptom management, may offer a path toward more effective and durable outcomes. The diversity of underlying mechanisms provides a wide range of potential therapeutic interventions (Table 2). Defining which of these approaches will ultimately prove effective for real-world treatment remains an open issue [100,101].

Although it is now well established that APS is a complement-mediated disease, this recognition is not yet fully reflected in therapeutic strategies. Clinical experience with complement inhibitors in APS remains limited, mostly involving CAPS cases. The efficacy of Eculizumab is supported by data from the international CAPS Registry, which reports complete remission in 74% of patients treated with a complement inhibitor [98]. This study is one of the largest examining the role of eculizumab in CAPS. Despite the relatively large sample size, given the rarity of CAPS, the retrospective design of the study and the lack of a control group might limit the generalizability of the findings. Its use in APS has shown promising results, providing a basis for further research on complement blockade as a therapeutic strategy in this syndrome. Currently, no data are available regarding the use of other complement inhibitors in CAPS. Ravulizumab, a long-acting C5 inhibitor, and Pegcetacoplan, a C3 inhibitor, as well as emerging oral proximal complement inhibitors, represent therapeutic options that warrant further investigation [90,102].

An ongoing clinical study is investigating the therapeutic potential of RAY121 in patients with immunological diseases. RAY121 is an inhibitor of the classical complement pathway, and the RAINBOW trial, a multicenter Phase 1b study, is assessing its safety and efficacy in patients with APS-associated nephropathy and/or livedoid vasculopathy [103]. Its results are eagerly anticipated. Moreover, the role of chimeric antigen receptor-T cell immunotherapy for APS is under investigation in several ongoing clinical trials [104]. In Table 1, the role of pharmaceutical agents targeting immunothrombosis in APS is summarized. Nevertheless, data regarding the role of these emerging therapies in APS do not come from randomized controlled trials, and more well-organized studies are crucial to better understand the role of these novel therapeutics.

We have to emphasize that the implementation of complement inhibition therapy in clinical practice might be challenging, mainly due to increased costs and possible risk of infections. However, careful selection of patients who might benefit from these therapeutics, possibly those with CAPS, APS-related thrombotic microangiopathy, and recurrent thrombotic events, along with meticulous infection prevention, with the essential vaccinations and antibiotic prophylaxis, when considered necessary, can help us to overcome these difficulties. Another possible problem in the use of complement inhibitors is the lack of biomarkers for patient selection and treatment outcomes assessment. Advances in other diseases, such as transplant-associated thrombotic microangiopathy, a complement-mediated disorder after hematopoietic cell transplantation, can serve as paradigms in the identification and use of possible diagnostic and prognostic complement biomarkers [105,106].

## 6. Conclusions

Although significant advances have been made in elucidating the pathophysiology of APS, it remains a complex clinical entity. Numerous pathogenic mechanisms have been proposed and studied, each contributing to the syndrome’s clinical heterogeneity. Complement activation, as previously discussed, plays a pivotal role in thromboembolic manifestations and actively drives the inflammatory cascade and immunothrombosis.

Despite the challenges, the pursuit of more effective therapies continues. The recently introduced 2023 ACR/EULAR classification criteria contribute significantly to this effort. The application of these criteria has been strongly encouraged among clinical investigators, as they promote the formation of more homogeneous patient cohorts, facilitating better clinical trial design. In parallel, advances in medical biotechnology have enabled the development and evaluation of novel therapeutic strategies.

While the application of these possible treatment options in the treatment of APS care is complex, ongoing research into its underlying mechanisms may reveal new targets and guide their appropriate use. Conducting clinical studies is essential to identify biomarkers that can guide the selection of therapeutic agents, determine optimal timing of administration, identify patient subgroups most likely to benefit, and inform combination strategies that maximize efficacy while minimizing adverse effects.

In all cases, effective management of APS requires a personalized approach to each patient. Looking ahead, a thorough evaluation of patients’ aPL profile, in combination with transcriptomic and epigenomic data analysis, may contribute to explaining the clinical heterogeneity of APS and lead to the development of more accurate prognostic and therapeutic protocols.

## Figures and Tables

**Table 1:** Summary of the 2023 ACR/EULAR classification criteria for APS.

Clinical Criteria	Laboratory Criteria
1. Venous thromboembolism	1. Lupus anticoagulant positivity
2. Arterial thrombosis	2. aPL test positivity
3. Microvascular manifestations	
4. Obstetric complications	
5. Manifestations from cardiac valves	
6. Thrombocytopenia	

APS: antiphospholipid syndrome, aPL: antiphospholipid antibodies.

**Table 2:** Pharmaceutical agents targeting immunothrombosis in APS.

Agent	Target	Pathophysiological Role
Adalimumab	TNFα antagonist	Reduces TF expression by monocytes
Rapamycin-Everolimus	mTOR pathway inhibitors	Prevent activation of monocytes and platelets
Dipyridamole	Antiplatelet agent	Reduces platelet hyperreactivity and oxidative stress
Ubiquinol	Antioxidant	Decreases reactive oxygen species
Anti-PSGL-1 mAb	Antibodies targeting P-selectin glycoprotein ligand-1	Suppress neutrophil adhesion to endothelium
Defibrotide	Oligonucleotides	Decrease thrombus size, NETs formation, and endothelial activation
Eculizumab	C5 complement inhibitor	Blocks the terminal complement pathway

APS: antiphospholipid syndrome; mTOR: mammalian target of rapamycin; NETs: neutrophil extracellular traps; TF: tissue factor; TNF-α: tumor necrosis factor-α.
